# Correspondence

**Published:** 1865-08

**Authors:** Ira D. Hopkins

**Affiliations:** Utica


### Correspondence.

UTICA, August 4, 1865.

To the Editor of the Buffalo Medical and Surgical Journal :

Sir:—I send you the following report of an autopsy, also a few facts connected with the case:

Autopsy July 27, 1865. On inspection of the body there was a lacerated wound on the right wrist; also two lacerated wounds on all of the fingers except the third, which had only one. On the left side of the head, just anterior to the meatus auditorious externus, was a lacerated wound, apparently having been produced by a gun-shot. On probing the wound, found that it extended into the cranium. Then removed the calvaria and exposed the brain, also removing it from the cranium. The ball entered the cranium through the petrous portion of the temporal bone, severing the carotid artery. The track of the ball was plainly indicated by a groove traversing the anterior portion of both hemispheres of the cerebellum. As it passed from the left lobe to the right lobe, it severed the anterior and middle portions of the medulla oblongata, just below the pons varolii. After leaving the right lobe it struck the right temporal bone, changed its course, going downwards, removing a small portion of the occipital bone on the right of the foramen magnum, and most likely passed into the spinal column.

The facts in the case are that he lived about five minutes, and walked about fifteen feet. This is as wonderful an exhibition of vitality, and the power of exercising volition and locomotion, as I ever saw recorded. I believe it is the opinion of all surgeons that severing any portion of the spinal marrow, above the third cervical vertebra, ordinarily causes instantaneous death. Please give your opinion as to the facts in the case, also of the writer's idea.

Yours, respectfully, IRA D. HOPKINS, M. D.

